# Unravelling personalized dysfunctional gene network of complex diseases based on differential network model

**DOI:** 10.1186/s12967-015-0546-5

**Published:** 2015-06-13

**Authors:** Xiangtian Yu, Tao Zeng, Xiangdong Wang, Guojun Li, Luonan Chen

**Affiliations:** School of Mathematics, Shandong University, Jinan, 250100 China; Key Laboratory of Systems Biology, Innovation Center for Cell Signaling Network, Institute of Biochemistry and Cell Biology, Shanghai Institutes for Biological Sciences, Chinese Academy of Sciences, Shanghai, 200031 China; Department of Respiratory Medicine, Zhongshan Hospital, Fudan University, Shanghai, China; Shanghai Institute of Clinical Bioinformatics, Fudan University Center for Clinical Bioinformatics, Shanghai, China; School of Life Science and Technology, ShanghaiTech University, Shanghai, 201210 China

**Keywords:** Gene expression, Expression variance, Precision medicine, Gene network, Network biomarker, Disease heterogeneity, Edge biomarker

## Abstract

In the conventional analysis of complex diseases, the control and case samples are assumed to be of great purity. However, due to the heterogeneity of disease samples, many disease genes are even not always consistently up-/down-regulated, leading to be under-estimated. This problem will seriously influence effective personalized diagnosis or treatment. The expression variance and expression covariance can address such a problem in a network manner. But, these analyses always require multiple samples rather than one sample, which is generally not available in clinical practice for each individual. To extract the common and specific network characteristics for individual patients in this paper, a novel differential network model, e.g. personalized dysfunctional gene network, is proposed to integrate those genes with different features, such as genes with the differential gene expression (DEG), genes with the differential expression variance (DEVG) and gene-pairs with the differential expression covariance (DECG) simultaneously, to construct personalized dysfunctional networks. This model uses a new statistic-like measurement on differential information, i.e., a differential score (DEVC), to reconstruct the differential expression network between groups of normal and diseased samples; and further quantitatively evaluate different feature genes in the patient-specific network for each individual. This DEVC-based differential expression network (DEVC-net) has been applied to the study of complex diseases for prostate cancer and diabetes. (1) Characterizing the global expression change between normal and diseased samples, the differential gene networks of those diseases were found to have a new bi-coloured topological structure, where their non hub-centred sub-networks are mainly composed of genes/proteins controlling various biological processes. (2) The differential expression variance/covariance rather than differential expression is new informative sources, and can be used to identify genes or gene-pairs with discriminative power, which are ignored by traditional methods. (3) More importantly, DEVC-net is effective to measure the expression state or activity of different feature genes and their network or modules in one sample for an individual. All of these results support that DEVC-net indeed has a clear advantage to effectively extract discriminatively interpretable features of gene/protein network of one sample (i.e. personalized dysfunctional network) even when disease samples are heterogeneous, and thus can provide new features like gene-pairs, in addition to the conventional individual genes, to the analysis of the personalized diagnosis and prognosis, and a better understanding on the underlying biological mechanisms.

**Electronic supplementary material:**

The online version of this article (doi:10.1186/s12967-015-0546-5) contains supplementary material, which is available to authorized users.

## Background

It is a challenging task to extract discriminative features from genes as relevant as possible for indicating different phenotypes [1], and in particular, these elaborately extracted features are expected to improve the understanding on complex diseases [2]. Gene expression analysis and gene network inference have been widely studied for extracting phenotype-related information in biological systems [3], but they are generally based on a group of samples with the same phenotype rather than a single sample, which prevents their applications to clinical data, e.g., disease diagnosis or prognosis on one sample from one individual. Therefore, how to infer discriminatively interpretable features of genes and their network in one sample is becoming an attractive and also urgent problem.

On one hand, conventional differential expression analysis of complex diseases requires the genes to have differential expressions between control and case samples, which is under the assumption that a gene in case samples would have consistent up-regulation or down-regulation than its expressions in control samples, or vice versa. But, recent studies indicate that many relevant (disease associated) genes are missed or hard observed from the analysis [4]. A key reason is that, different from the previous assumptions, the disease samples tend to be in different sub-clones, stages or subtypes, which result in heterogeneous expression patterns. Under this complicated situation, some genes would show up-regulation in a part of disease samples but down-regulation in the other part of disease samples, which are non-consistently compared to control samples (e.g., heterogeneity of diseases [5]). These genes are always rejected by the significance test in the conventional differential expression analysis. Thus, the first important task is how to carefully select feature genes and gene-pairs for deep disease studies in a network manner. Particularly, analyzing the differential expression variance of genes (i.e., nodes of a gene network) and differential expression covariance of gene-pairs (i.e. edges of a gene network) is expected to be able to effectively extract the informative gene features of network [4], which improves the interpretability of network features.

On the other hand, the differential gene expression analysis can be applied to a group of samples (e.g. *T* test used) or a single sample (e.g. fold-change used). Meanwhile, the expression variance of a gene or expression covariance of a gene-pair is a statistic on samples or populations. These two kinds of features of gene expression or gene network are usually used on multiple samples rather than one sample. However, in clinical practice on cancer diagnosis or treatment [6], only one sample is usually available for each patient [7]. For example, there is one sample (e.g., a sample from blood drawn) obtained in the physical examination when diagnosing some suspected victims or onset patients; or, a sample will be collected at a planed time after surgery when taking the follow-up of therapy-treated patients. Under these biological or physical constraints in actual situation, the second important task is to elaborately select feature genes and their network in a single-sample manner, for improving the discriminative ability by considering personalized characteristics.

**Figure 1:**
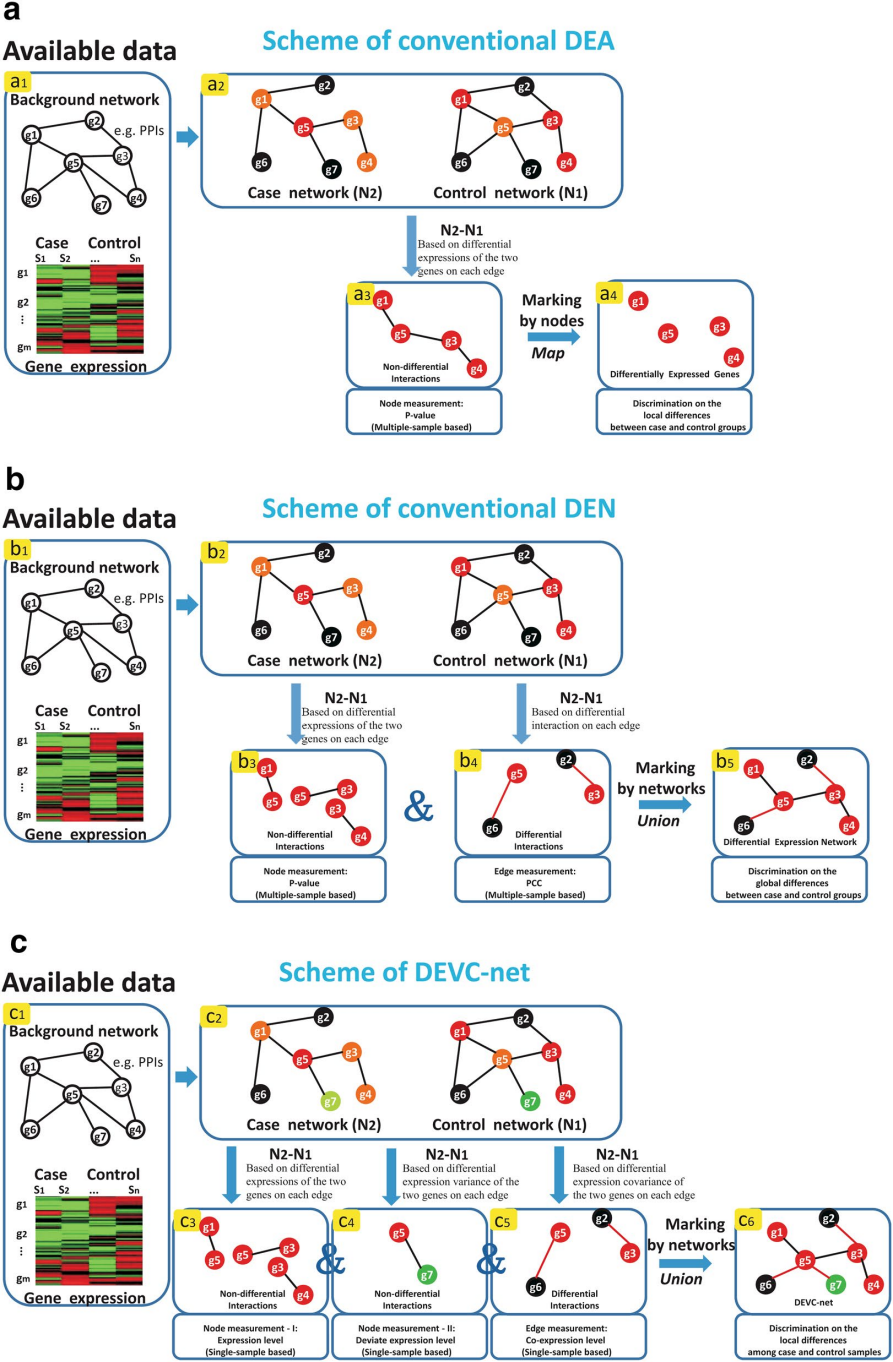
Overview of DEVC-net on extracting discriminatively interpretable features of a gene network by combining gene expression, and expression variance/covariance. **a** The framework of conventional differential expression analysis (DEA). Only differential expression is considered in the conventional DEA, which can be estimated in a multiple-sample manner (e.g., P-value from statistic test) or in a single-sample manner (e.g., fold-change). **b** The framework of conventional differential expression network (DEN). In the conventional DEN, the information of differential expression variance has not been considered. **c** The framework of the proposed DEVC-net. Compared to the conventional network-based approaches, DEVC-net has two advantages: one is to use differential expression variance and the other is to design the measurements of differential expression variance/covariance in a single-sample case. Obviously, DEVC-net can be easily applied in a multiple-sample case. Note that, the gene is labeled in *red* if it has differential expression between case or control, and in *green* if has differential expression variance; The gene is labeled in *black* if there is no significant difference between case and control; The gene pair is labeled in *red* if the two genes have differential expression covariance, otherwise *black*; Besides, PPI means protein–protein interaction, and PCC means Pearson correlation coefficient.

To address the above two problems together, a novel differential network model is proposed to integrate Differential gene Expression, differential expression Variance and differential expression Covariance by a differential score DEVC. DEVC-net (DEVC-based differential expression network, and see Figure 1c) can be constructed for groups of patients by the divergent differential expression and network features, and also rebuilt for each patient as the personalized dysfunctional gene network.

Note that, as basic elements of DEVC-net, the gene-pairs rather than individual genes are generalizable to cases of biomarkers or other biological signatures. Firstly, an important evidence of gene-pair (e.g. edge or interaction) signatures is the discovery of ‘edgetics’ diseases, and the study of ‘edgetics’ also revealed the malfunctions of interactions [8] as the key molecular mechanisms relevant to complex diseases. Secondly, by a data-driven method, the concept of the expression reversal of gene-pairs has been used to identify putative determinants (e.g. toggle-switch circuits) of cell fate [9], which reveals gene-pair expression signatures of lineage control. Thirdly, although there are many underlying biological processes (e.g. transcriptional factors, regulatory genes, etc.) that can modulate the gene-pairs, these regulatory elements are usually not significant enough to be biomarkers or signatures due to biological natures or limits of bio-technology. For example, the network-based activity of TP53 rather than original expression can correctly indicate the disease status and treatment status [10]; and from non-differentially expressed genes, many gene-pairs have been found to display significantly differential expression correlations [4], although the regulatory mechanism behind them are still unclear or hard detected. All these facts suggest that the gene-pair based approach (i.e. DEVC-net) is actually necessary and suitable in disease study or other general phenotype study, in addition to the conventional gene-based methods.

A proof-of-concept study of DEVC-net has been mainly conducted on the investigation of prostate cancer. Firstly, we show that the differential network has a new bi-coloured topological structure, characterizing the global expression changes between normal and diseased samples. DEVC-net has a sub-network that is mainly composed of genes/proteins controlling various biological processes, and particularly displays a non hub-centred structure in keeping with the pathway structure. Secondly, by compared to genes with differential expression used in the traditional methods, the genes with the differential expression variance or gene-pairs with the differential expression covariance are shown to be new informative sources of local expression changes of a given patient, and can be used to identify discriminative genes and gene-pairs which are ignored previously. More importantly, DEVC-net quantitatively measures the expression levels or activities of different kinds of feature genes and their network or modules in one sample, which cannot be obtained in a traditional way. In particular, we found a significant differential module including genes/proteins with alternative splicing functions, which is known as a key factor of the heterogeneity of prostate cancer. Therefore, DEVC-net indeed has clear advantages to effectively extract discriminatively interpretable features of gene/protein network for one sample, e.g., personalized dysfunctional gene network, even when disease samples are heterogeneous. Thus, DEVC-net can provide new features like gene-pairs, in addition to individual genes, to the analysis of the personalized diagnosis and prognosis from the perspective of systems medicine or precision medicine, and a better understanding on the underlying biological mechanisms (Additional files 1, 2, and 3).

## Methods

The DEVC-net (Figure 1c) is proposed to model the differential expression patterns among different samples with particular phenotypes (e.g., dissimilar patients) by integrating genes with the differential expressions (DEG), genes with the differential expression variances (DEVG) and gene-pairs with the differential expression covariances (DECG). Firstly, three measurements are designed to evaluate differential information: (1) the original expression level indicating DEG; (2) the absolute relative expression level indicating DEVG; and (3) the co-expression level indicating DECG. Secondly, a differential score (DEVC) based on such divergent differential information is proposed to quantify the differential network/module. Then, a novel bi-coloured differential expression network, i.e. DEVC-net, can be constructed for groups of patients. The genes of DEG and DEVG stand for two kinds of nodes in the differential expression network (DEN) [11], and the gene-pairs of DECG are a group of edges in the network.

Obviously, the new numerical measurement DEVC can discriminatively quantify the expression state of different kinds of feature genes and their network in one sample, and DEVC-net can thus provide interpretable clues of diseases as a personalized dysfunctional gene network for each individual. Note that, the DEVC-net demands the case/control cohorts (e.g., each cohort should have at least two samples which ensure the availability of the estimated statistical values of the transcripts) although it would be difficult on rare diseases. All details are given as follows.

It should be emphasized that the DEVC-net mainly focuses on the extraction of novel features on gene network level to characterize the disease, especially the disease state of individuals. By DEVC-net, we can obtain at least four kinds of features: the conventional genes with the differential expression; the new genes with the differential expression variance; the new gene-pairs with the differential expression covariance; and the new network module combined of the above three kinds of feature genes. In addition, the numerical measurements for these four kinds of features are also proposed and evaluated. Therefore, similar to the DEGs used in the traditional works, such output of DEVC-net can also be directly used in diagnosis and prognosis as quantitative criteria. In fact, DEVC-net exploits additional new information (e.g., absolute relative expression level and co-expression level) rather than only the expression level to identify new feature genes (e.g., DEVG and DECG), which can better separate case and control groups. Therefore, DEVC-net is actually a robust collection of feature genes (e.g., potential biomarker genes or gene-pairs). For a test sample to make a diagnosis, one only needs to identify the genes with particular differential expression features based on the corresponding measurements (i.e., original expression level for DEG, absolute relative expression level for DEVG, co-expression level for DECG, and even differential score for differential module), and compare these genes or gene-pairs with the ones comprising the differential network.

To evaluate these new features derived from DEVC-net, we have conducted a proof-of-concept study on real disease data: (1) We compared DEG and DEVG on discriminating/clustering disease samples by different numerical measurements, which demonstrates that the combination of DEG and DEVG with their corresponding measurement has better performance (significance evaluated by P-value) than themselves (see the detail comparison study between DECG and DEG in previous work [4]); (2) Based on network modules, we further compared different combinations of DEG, DEVG and DECG, and found that the best performances (significance evaluated by P-value) were achieved when all three kinds of feature genes were combined together, which supports that DEVG and DCCG are meaningful and complementary to the conventional DEG; (3) Furthermore, a representative network module is illustrated with DEG, DEVG and DECG, and their expression patterns in individual patients, which reveals the dysfunctional individual network; (4) As an important biological mechanism associated to such a representative network module, alternative splicing related to module genes is discussed in an independent dataset. In all, in addition to the individual genes, DEVC-net can provide new features like gene-pairs to the analysis of the personalized diagnosis and prognosis, and a better understanding on the underlying biological mechanisms. As one future work, we will apply the general classification or prediction model, e.g., logistic regression or decision tree, to learn/train these new features for diagnosis and prognosis by balancing the sensitivity and specificity of disease test.

The analysis approach of DEVC-net has been implemented as a package of Matlab scripts, and alternative R scripts will be available in near future. All codes can be requested from the authors.

### Differential score based on differential expression, variance and covariance (DEVC)

A few notations are defined for convenience. For an expression network or a module, it has a node (gene) set *V* and an edge (gene-pair) set *E*; and a sample set is *S* including all control and case samples. The expression of gene *n* is e_n_. Meanwhile, the sign of the regulation trend of gene *n* is sign(*n*) which is +1 when this gene is up-regulated and −1 when this gene is down-regulated; and the sign of the regulation trend of interacted genes *m* and *n* is sign(*m*, *n*) which is +1 when these two genes’ expression covariance/correlation increases and −1 when expression covariance decreases.

#### Differential gene expression

Given a gene *x* that has expression profiles in control samples as *X* and in case samples as *X*′, the expression variance of this gene in control condition is E((X − *u*)^2^) and in case condition is E((X′ − *u*′)^2^). Here, *u* and *u*′ are means of the expressions of gene *x* in control and case samples, respectively. Then, the conventional criterion and measurement of a gene with differential expression (DEG) are:

1 $$ {\text{H}}_{0}:{\text{ E}}\left( X \right) = {\text{E}}\left( {X^{\prime}} \right);{\text{ H}}_{0} \;{\text{rejected}} $$

where *X* or *X*′ is the original expression level, e.g., $e_{n}^{s}$ represents the expression of a gene *n* in a sample *s* from sample set *S*.

#### Differential expression variance

Differential expression of a gene requires the gene’s expressions under different conditions to distribute around different mean expression levels. Meanwhile, differential expression variance can be defined as the distance between a gene’s original expression level and its mean expression level (e.g., deviation) that are significantly different under different conditions, such as:

$$ {\text{H}}_{0}:{\text{ E}}\left( {|X - u|} \right) = {\text{E}}\left( {|X^{\prime} - u^{\prime}|} \right);{\text{ H}}_{0} \,{\text{rejected}} $$

Notice that how to measure the differential expression variance in one sample is one difficult problem. For example, it cannot or is hard to determine which expression mean of *u* and *u*′ would be used to test the expression of a test sample. However, given a few genes in non-DEGs with the same *u* and *u*′, this set of genes can be quantified in one sample by the distances from their expression values to the same prior-estimated mean expression level. Therefore, the criterion and measurement of a gene with the differential expression variance (DEVG) for one sample analysis are:

2 $$ {\text{H}}_{0}:{\text{ E}}\left( {|X - u|} \right) = {\text{E}}\left( {|X^{\prime} - u^{\prime}|} \right);{\text{ H}}_{00} :{\text{ E}}\left( X \right) \ne {\text{E}}\left( {X^{\prime}} \right);{\text{ H}}_{0} \;{\text{and H}}_{00} \;{\text{rejected}} $$

where *X* or *X*′ is the original expression level, meanwhile |*X* − *u*| or |*X*′ − *u*′| is the absolute relative expression level, e.g., $|e_{n}^{s} - \frac{{\sum\limits_{\tau \in S} {e_{n}^{\tau } } }}{|S|}|$ represents the absolute relative expression of a gene *n* in a sample *s* from sample set *S*.

Actually, given *X* or *X*′ satisfying normal distribution, |*X* − *u*| or |*X*′ − *u*′| will be folded normal distribution. Then the Wilcoxon rank sum test instead of Student’s T-test is used in significance test to reject or accept the null hypothesis.

#### Differential expression covariance

Given two genes (*x* and *y*) that have expression profiles in control samples as *X* and *Y* and in case samples as *X*′ and *Y*′, the expression covariance of these two genes in control condition is E((X − *u*)(Y − *v*)) and in case condition is E((X′ − *u*′)(Y′ − *v*′)). Here, the *u* and *u*′ are the means of the expressions of gene *x* in control and case samples, respectively; meanwhile the *v* and *v*′ are the means of the expressions of gene *y* in control and case samples, respectively. The expression covariance between two genes will have a significant change when E((X − *u*)(Y − *v*)) and E((X′ − *u*′)(Y′ − *v*′)) are non-equivalent. Thus, the co-expression level *C* of a gene-pair (*x* and *y*) is introduced as the product of these two genes’ normalized expression in one sample, e.g., *C* just equals (X − *u*)(Y − *v*) in control condition and C′ is (X′ − *u*′)(Y′ − *v*′) in case condition. This roughly gives a criterion to judge the differential expression covariance of a gene-pair (the involved gene is DECG, e.g., gene with the differential expression covariance): the co-expression value of a gene-pair is significantly different in control and case conditions, e.g., E(*C*) = E(*C*′) rejected.

Obviously, the co-expression level can be conveniently used to support the conventional differential network analysis on multiple samples by indicating the differential correlation of a gene-pair under different conditions, but, it still has the difficulty to measure the differential gene-pairs in one sample [4]. This is because the average expressions of a gene *x* (or gene *y*) under control and case conditions are generally different (e.g., *u* ≠ *u*′), and thus, it cannot determine which estimated mean expression level *u* and *u*′ (or *v* and *v*′) would be used to normalize the expressions of a test sample. Using a strategy similar to the above DEVGs, we can find two special sub-sets of gene-pairs to make full use of differential expression covariance in single samples. One set contains gene-pairs whose two genes have differential covariance but both do not have significant differential expressions (i.e., *u* = *u*′ = u*, and *v* = *v*′ = v*), and obviously this kind of gene-pairs can uncover new genes missed in the conventional differential expression analysis. The other set has gene-pairs whose two genes have differential expression covariance and differential expression but satisfy: E((X − u*)(Y − v*)) = E((X′ − u*)(Y′ − v*)) rejected by the significance tests, where u* is the mean of the expressions of gene *x* in all control and case samples and v* is the mean of the expressions of gene *y* in all samples. Thus, for a test sample, its expressions can be normalized by the estimated u* and v*. Therefore, the criterion and measurement of a gene-pair (DECG) for one sample analysis is:

3 $$ {\text{H}}_{0}:{\text{ E}}\left( {\left( {X - u^*} \right)\left( {Y - v^*} \right)} \right) = {\text{E}}\left( {\left( {X^{\prime} - u^*} \right)\left( {Y^{\prime} - v^*} \right)} \right);{\text{ H}}_{0} \;{\text{rejected}} $$

where *X* − u* or *X*′ − u* (*Y* − v* or *Y*′ − v*) is the relative expression level, *C* = (*X*−u*)(*Y* − v*) or *C*′ = (*X*′ − u*)(*Y*′ − v*) is the co-expression level, e.g., $(e_{m}^{s} - \frac{{\sum\nolimits_{\tau \in S} {e_{m}^{\tau } } }}{|S|})(e_{n}^{s} - \frac{{\sum\nolimits_{\tau \in S} {e_{n}^{\tau } } }}{|S|})$ represents the co-expression level of a gene pair between two genes *m* and *n* in a sample *s* from sample set *S*.

Actually, given X, X′, Y, or Y′ satisfying the normal distribution, (X − u*)(Y − v*) or (X′ − u*)(Y′ − v*) will be normal product distribution [12], and thus, the Wilcoxon rank sum test instead of Student’s T-test is used in significance test to reject or accept the null hypothesis.

#### Differential score (DEVC)

Based on the above measurements for one gene’s expression, one gene’s expression variance and two genes’ expression covariance in individuals (as formula 1–3), an additive score DEVC is designed to measure the differential expression of a group of genes as a network or sub-network/module in one sample (Note that, the additive score is a common strategy to measure the expression status or activity of network/module [13, 14]). The measurement of differential expression for a sub node-set DEG is mDEG (formula 4); the measurement of differential expression variance of a sub node-set DEVG is mDEVG (formula 5); the measurement of differential expression covariance of a sub edge-set DECG is mDECG (formula 6); thus, the integrative measurement of the differential expression of whole network is differential score DEVC calculated as formula 7. In formula 4–7, *V* represents a set of nodes/genes; *E* represents a set of edges/gene-pairs; *S* represents a set of all samples; and *s* represents a particular sample. Therefore, such four formula calculate different measurements/scores on nodes/genes and/or edges/gene-pairs on one sample, respectively.

4 $$ mD{\text{EG}}(V,E,s) = \sum\limits_{n \in V,n \in DEG} {\text{si} gn(n)e_{n}^{s} } $$

5 $$ mDEVG(V,E,s) = \sum\limits_{n \in V,n \in DEVG} {\text{si} gn(n)|e_{n}^{s} - \frac{{\sum\limits_{\tau \in S} {e_{n}^{\tau} } }}{|S|}|} $$

6 $$ mDECG(V,E,s) = \sum\limits_{(m,n) \in E,(m,n) \in DECG} {\text{si} gn(m,n)\left(e_{m}^{s} - \frac{{\sum\limits_{\tau \in S} {e_{m}^{\tau} } }}{|S|}\right)\left(e_{n}^{s} - \frac{{\sum\limits_{\tau \in S} {e_{n}^{\tau} } }}{|S|}\right)} $$

7 $$ DEVC(V,E,s) = mDEG(V,E,s) + mDEVG(V,E,s) + mDECG(V,E,s) $$

Note that, for a single score like mDEG/mDEVG/mDECG, a network with more nodes tends to have a higher score value, and thus, it is necessary to include a normalization term (1/k or 1/sqrt(k) where k is the number of nodes or edges in this network) because there is a possibility to compare networks with different number of nodes, especially in those fields like network decomposition or sub-network extraction [15]. However, in our work, we use the three measurements (i.e., formula 4–6) to evaluate the same network in different conditions (e.g., samples) rather than network comparison, so that the normalization term is not necessary here. In addition, if including the normalization terms, the combined score DEVC would be changed as a weighted form defined in formula 7, which is worthy of careful study in future.

### Differential expression network quantified by differential score (DEVC-net)

Particularly, DEVC can enhance the differential expression network (DEN) [11], which models differentially expressed genes as nodes and differentially correlated gene-pairs as edges on the network level. The so-called DEVC-net (Figure 1c) rather than DEN (Figure 1b) can analyse and measure differential expression of genes and gene-pairs in one sample simultaneously. The construction of DEVC-net includes the following three steps, which assumes to have a background network (e.g., PPI network) and expression data for case and control (e.g., disease and normal) samples.

1. Extracting DEVC-based differential interactions (Step c5 in Figure 1c): a gene pair as edge from a background network, e.g., PPI network, is selected only if its corresponding two genes have significant differential expression covariance (e.g., for DECG, the P value of Wilcoxon rank sum test for significance on the co-expression level between case and control samples is no larger than 0.05).
2. Extracting DEVC-based non-differential interactions (Step c3 and c4 in Figure 1c): a gene pair from a background network is selected only if its corresponding two genes both have significant differential expression or differential expression variance (e.g., for DEG, the P value of T-test significance on the original expression level between case and control samples is no larger than 0.05; for DEVG, the P value of Wilcoxon rank sum test on the absolute relative expression level between case and control samples is no larger than 0.05).
3. Constructing the DEVC-based differential expression network (DEVC-net in Step c6 in Figure 1c): The union of aforementioned two kinds of interactions can construct a novel differential expression network, which is able to characterize the alterations of genes’ expression, expression variance and expression covariance among case and control samples simultaneously.

## Results

### A proof-of-concept study of DEVC-net on real gene expression datasets

As a proof-of-concept study of DEVC-net on complex diseases, we mainly carried DEVC-net analysis on the investigation of prostate cancer [16]. The gene expression dataset of prostate cancer was downloaded from NCBI GEO [17] with access ID GSE6099 [16]. It contains 84 tissue samples with 8247 genes after pre-procession. This is a benchmark in feature study [18]. These previous researches focus on the differential expressions of individual genes. By contrast, DEVC-net can discover those genes with differential expression variance or gene-pairs with differential expression covariance in one sample on the differential network level, which are generally previously disregarded. Specifically, we design an analysis and evaluation framework as follows:

1. Selecting genes with the differential expression (DEGs); genes with the differential expression variance (DEVGs); and gene-pairs with the differential expression covariance (DECGs). To select DEGs or DEVGs, the P-value of the significance of differential expression or differential variance is calculated and ranked from the least to the largest, and the Top-ranked N genes are chosen (where N is set to 1000 as the same as the previous study [18]). Match these genes with known disease genes from GeneCards database [19].
2. Constructing DEVC-net and obtain differential modules by MCL [20], where MCL has only one parameter I (inflation), which is set as 1.8 according to the empirical value [21, 22]; Note that, MCL algorithm (Markov Clustering) is a conventional network (module) decomposition method [20], designed specifically for simple graphs (e.g., only network topology focused) and weighted graphs (e.g., both network topology and biological significance focused), whose basic assumption is that random walks on a graph will infrequently go from one natural cluster to another depending on estimated graph transition probability; Analyzing the network centralities of global and local topological structures of DEVC-net, e.g., closeness and betweenness [23, 24] or graph entropy [25, 26].
3. Measuring the expression state of differential modules in each sample by differential score DEVC and it’s several components; Use the quantified modules as new features to recognize disease samples from normal ones.

Based on the selected genes and their measurements (e.g., expression level of DEGs or absolute relative expression level of DEVGs), the samples can be clustered into two groups (22 samples in the early stage v.s. 62 samples in the advanced stage [16]) by K-means. We run K-means on these genes’ corresponding expression profiles by 1,000 times to avoid the bias in K-means analysis and the influence of parameters. And the accuracy of K-means is used to evaluate the efficiency of the extracted gene features. Given the known samples in *n* different phenotypes that are:

$$ \{ S_{i} \}_{i = 1}^{n} $$

While the gene clustering gives *m* gene clusters corresponding to *m* candidate phenotypes:

$$ \{ C_{j} \}_{j = 1}^{m} $$

Then, the identification accuracy, or the efficiency of extracted gene features, is calculated as:

$$ A = \frac{{\mathop {\hbox{max} }\limits_{\tau ([1,m])} \sum\limits_{j = 1}^{m} {|C_{j} \cap S_{\tau (j)} |} }}{{\sum\limits_{i = 1}^{n} {|S_{i} |} }} $$

where $\tau :[1,m] - > [1,n]$ is any map function. Obviously, the selected genes are the main factors to determine the downstream analysis performance, and the accuracy is used for performance evaluation.

Besides, a toy model has been given to show the conventional features and our new ones in a simulated data with heterogeneous expression patterns (Figure S1), and the evaluations are also given on other datasets related to diabetes [27]. All these additional results can be seen in the supplementary files (Additional files 1, 2, and 3).

### Bi-coloured structure of dysfunctional gene network revealed by DEVC-net

**Figure 2:**
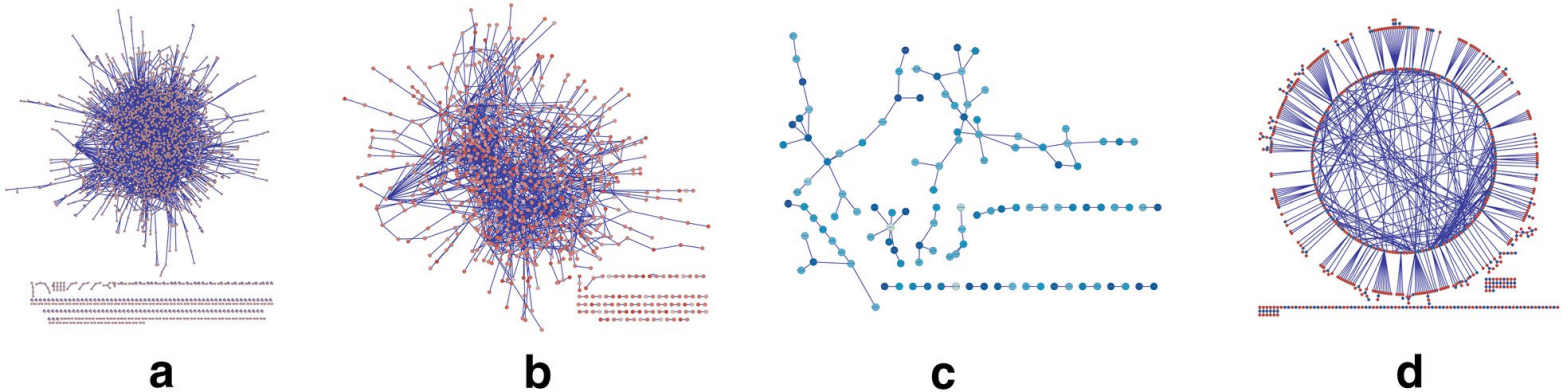
Topological structures of DEVC-net and its sub-networks. **a** DEVC-net. **b** DEG-subnet, i.e., DEGs induced sub-network, which uses only DEGs from DEVC-net as nodes. **c** DEVG-subnet, i.e., DEVGs induced sub-network, which uses only DEVGs from DEVC-net as nodes. **d** DD-subnet, i.e., DEGs & DEVGs induced bipartite sub-network, in which every edge connects one DEG and one DEVG.

Different from conventional DEN [11], DEVC-net shows a bi-coloured topological structure, which consists of one set of nodes representing DEGs and the other set of nodes representing DEVGs. Notice that there are a few genes as DECGs but they have no differential expressions on genes/nodes. Focusing on genes/nodes, the DEGs induced sub-network in DEN (i.e., DEG-subnet using DEGs as nodes), the DEVGs induced sub-network (i.e., DEVG-subnet using DEVGs as nodes), and DEGs & DEVGs induced bipartite sub-network (i.e., DD-subnet using edge to connect one DEG and one DEVG) are further investigated from the viewpoint of network centrality [24] of their topological structures. Notice that, these induced sub-networks are all based on the prior-known protein interaction networks [28]. Table 1 shows a significant characteristic of such a bi-coloured differential expression network: its component DEVG-subnet has the largest degree centrality but the least closeness centrality, compared to the global or other local network structures. This phenomenon indicates that the interactions among DEVGs prefer to link as a path rather than hub-centred structure in a general biological network (Figure 2c), which means that DEVGs would have long-term interactive pattern to achieve complicated control mechanism on a biological network involved in disease development and progression. Although DD-subnet has a particular bipartite topological structure (Figure 2d), its many centralities are similar to those of global DEVC-net (Figure 2a) or local DEG-subnet (Figure 2b), and thus this kind sub-network is still hub-centred. Besides, this characteristic of topological structure of DEVC-net has also been observed in the additional analysis on diabetes in supplementary files.

**Table 1:** The comparison of network centrality among different sub-networks of DEVC-net on prostate cancer dataset

	# Node	# Edge	Degree	Closeness	Betweenness	Entropy
DEVC-net	1,836	3,182	0.00189	0.15908	0.00163	6.90187
DEG-subnet	889	1,582	0.00400	0.17885	0.00323	6.23422
DEVG-subnet	112	95	0.01528	0.03202	0.00334	4.55011
DD-subnet	641	790	0.00385	0.11991	0.00467	6.03607

As known, the degree centrality, or most other network centralities usually indicate an average effect. The high degree centrality means many nodes in a network would have high degree. By contrast, hub-centred structure expects only one or very few nodes with extremely high degree than others. In our experimental case, that means it is possible no one or so many nodes with extremely high degree than others, i.e. no node can be thought as a hub with significance. In addition, a simple example about such relation between degree centrality and hub-structure have been illustrated and discussed in supplementary document.

### New informative sources of disease genes and gene-pairs extracted by the features of differential expression variance/covariance

Table 2 illustrates that, for the selected similar number of different gene features (e.g., top-1000 DEGs or DEVGs here), the original expression level of DEGs (DEG_ori) is naturally better than those of DEVGs (DEVG_ori) because the average accuracy of the sample-clustering based on DEGs (DEG_ori) for 1000 times is significant higher than that based on DEVGs (DEVG_ori), where P-value of T-test approaches zero. By contrast, the absolute relative expression levels of DEVGs (DEVG_rel) are better than those of DEGs (DEG_rel), i.e., the P-value of T-test on the clustering accuracies for 1,000 times is close to zero too. This supports that the differential variances of genes are indeed important to indicate phenotypes even though these genes would not be differentially expressed in the conventional analysis. It also indicates that the absolute relative expression level would be an appropriate measurement of DEVGs in a single sample. Particularly, the simple combinations of such two kinds of genes (DEG_ori & DEVG_rel, e.g., the combined top-500 DEGs and top-500 DEVGs here) tend to achieve the best performance (i.e., this combined feature has the largest average accuracy is significant compared to other features), and thus DEVG and DEG would be complementary kinds of gene features, and capture the differential expression and differential expression variance for genes, respectively.

**Table 2:** The comparison on DEG and DEVG with particular measurements on prostate cancer dataset

Methods*	DEG_ori	DEG_rel	DEVG_ori	DEVG_rel	DEG_ori & DEVG_ori	DEG_ori & DEVG_rel
Mean of accuracy	0.7803	0.5825	0.5965	0.6262	0.7592	*0.8871*
Std of accuracy	0.0309	0.0520	0.0229	0.0217	0.0375	0.0918

Italic value indicates the best performance in method comparison.

* DEG_ori means that we selected genes with differential expression as features, and the original/raw expression values as measurements of these conventional features used in the sample-clustering evaluation; Meanwhile, DEG_rel means that we selected genes with differential expression as features, but the proposed absolute relative expression values as measurements of these conventional features. Similarly, DEVG_ori means that we selected novel genes with differential expression variances as features, but the original/raw expression values as measurements of these new features; DEVG_rel means that we selected novel genes with differential expression variances as features, and absolute relative expression values as suitable measurements of these new features. There are six strategies evaluated, and each strategy applied particular feature genes and corresponding measurements for sample-clustering. For each strategy, the sample–clustering has been rerun 1,000 times, and the mean and variance of accuracies are the final performance of such a strategy.

The enrichment of the known disease associated genes from GeneCards database [19] provides additional evidence that genes with differential expression variance are also effective to catch the potential pathogen mechanism. Totally, 1661 prostate cancer related genes were extracted from GeneCards; and 188 DEGs in Top-1000 (P = 0.8615, which is calculated by hypergeometric test with the population as the above pre-processed 8247 genes, and the same in bellows) were found to be prostate cancer associated, while 225 DEVGs in Top-1000 (P = 0.0223) were detected. Thus, in addition to the conventional DEGs, new gene features (e.g., DEVGs) would lead to effective disease gene identification.

The DECGs (i.e., the genes from differentially correlated gene-pairs in the previous edge biomarker study [4]) also represent complementary gene expression information (e.g., discriminate information in non-differentially expressed genes), and the feature of expression covariance also represents new information [4]. In the analysis of DEVC-net, the original expression level of DEGs, absolute relative expression level of DEVGs, and co-expression level of DECGs are used respectively by default.

### Advanced discrimination on phenotypes indicated by the quantified personalized dysfunctional gene network and module

In addition to individual genes with the differential expressions, DEVC-net provides a new expression-weighted (differential) sub-network [29] describing malfunctions of a biological system in diseases. Although conventional differential network analysis [11, 29–31] is limited to indicate the network differences between groups of samples (e.g., normal and disease samples), DEVC-net can further indicate the network differences among individual samples by the personalized dysfunctional gene network, and thus, it can enhance the phenotype identification, e.g., disease diagnosis or prognosis.

DEVC-net can be decomposed into differential modules by MCL approach as shown in Table S1. Based on these modules, the differential scores (e.g., activities of modules) instead of expression level of single genes are used to cluster samples. Compared to the conventional module-based methods, the differential score DEVC (mDEG + mDEVG + mDECG) and its six kinds of components have been respectively used to classify the binary phenotypes, e.g., normal and prostate cancer samples.

In Table 3, the clustering performances demonstrate that: (1) the differential information involved in DEVGs or DECGs has observable discrimination ability on phenotype identification, although it is not better than the conventional DEGs when these different gene features are separately used to measure differential modules; (2) the combination of different gene features on quantifying network modules effectively promotes the clustering performance, particularly, the clustering accuracy achieves the largest and most robust when combining DEGs, DEVGs and DECGs together, e.g., DEVC score.

**Table 3:** The comparison on different combinations of feature genes of DEVC-net on prostate cancer dataset

Methods*	DEG	DEVG	DECG	DEG & DEVG	DEG & DECG	DEVG & DECG	DEG & DEVG & DECG
Mean of accuracy	0.8333	0.5800	0.6831	0.8571	0.8452	0.6359	*0.8631*
Std of accuracy	0.0357	0.0460	0.1481	0.0119	0.0238	0.1003	0.0060

Italic value indicates the best performance in method comparison.

* DEG means that we used only mDEG score (formula 4) to measure modules and applied these quantified modules for sample-clustering; DEVG means that we used only mDEVG score (formula 5); DECG means that we used only mDECG score (formula 6); DEG & DEVG means that we used the combination of DEG and DEVG; DEG & DECG means that we used the combination of DEG and DECG; DEVG & DECG means that we used the combination of DEVG and DECG; DEG & DEVG & DECG means that we used the combination of all, i.e., DEVC score (formula 7). For each combination, the sample–clustering has been rerun 1000 times, and the mean and variance of accuracies are the final performance of such a strategy.

**Figure 3:**
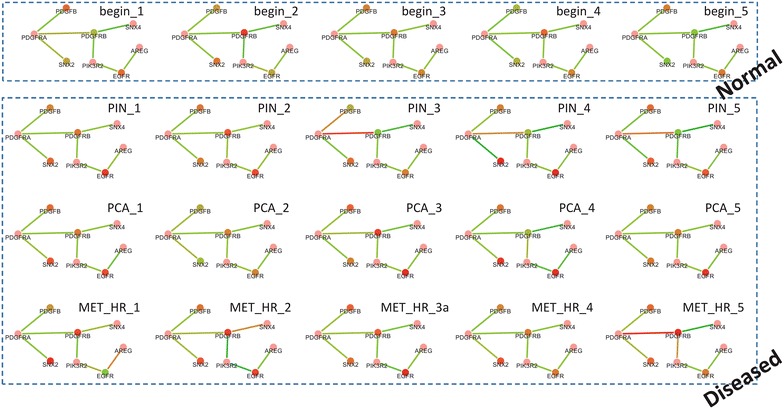
Personalized dysfunctional gene networks based on differential module related to prostate cancer. This module has its significant enrichment on KEGG prostate cancer pathway. It includes four DEGs as PDGFRB, PDGFB, SNX2, EGFR, and seven gene-pairs of DECGs as (PDGFRA, PDGFRB), (SNX4, PDGFRB), (PDGFB, PDGFRA), (SNX2, PDGFRA), (PDGFRB, PIK3R2), (EGFR, PIK3R2), (EGFR, AREG). The personalized network structures of this module have been displayed on five normal samples and other 15 disease samples, in which nodes with *red*/*green* colour represent genes with significantly high/low expression level, and edges with *red*/*green* colour represent gene-pairs with significantly high/low co-expression level.

To illustrate the personalized dysfunctional networks/modules for individual patients and their ability on disease classification, a number of representative examples are shown in Figure 3. A prostate cancer related module was investigated (due to its significant enrichment on KEGG prostate cancer pathway), which has DEGs as PDGFRB, PDGFB, SNX2, EGFR and DECGs as (PDGFRA, PDGFRB), (SNX4, PDGFRB), (PDGFB, PDGFRA), (SNX2, PDGFRA), (PDGFRB, PIK3R2), (EGFR, PIK3R2), (EGFR, AREG). Its personalized network structures for five normal samples and other 15 disease samples are displayed in Figure 3. Nodes with red/green colour represent genes with significantly high/low expression level; edges with red/green colour represent gene-pairs with significantly positive/negative co-expression. Obviously, the DEGs as PDGFB, SNX2, EGFR can discriminate many normal and disease samples, e.g., these genes tend to have high expression levels for the same patients. A few samples (PIN_3, PCA_2, MET_HR_1) seem not to satisfy this rule on the expression pattern, however, they have other possible discriminative features on edges: (PDGFB, PDGFRA) have high co-expression in PIN_3 or PCA_2 but not in other normal ones; (SNX4, PDGFRB) or (EGFR, AREG) have high co-expression in MET_HR_1 but not in other normal ones. Thus, this example strongly explains the rationality of combining multiple differential expression patterns for distinguishing individual patients, e.g., reconstructing the personalized dysfunctional gene networks/modules.

### Alternative splicing as the key factor of disease heterogeneity unravelled by a significant differential module

**Figure 4:**
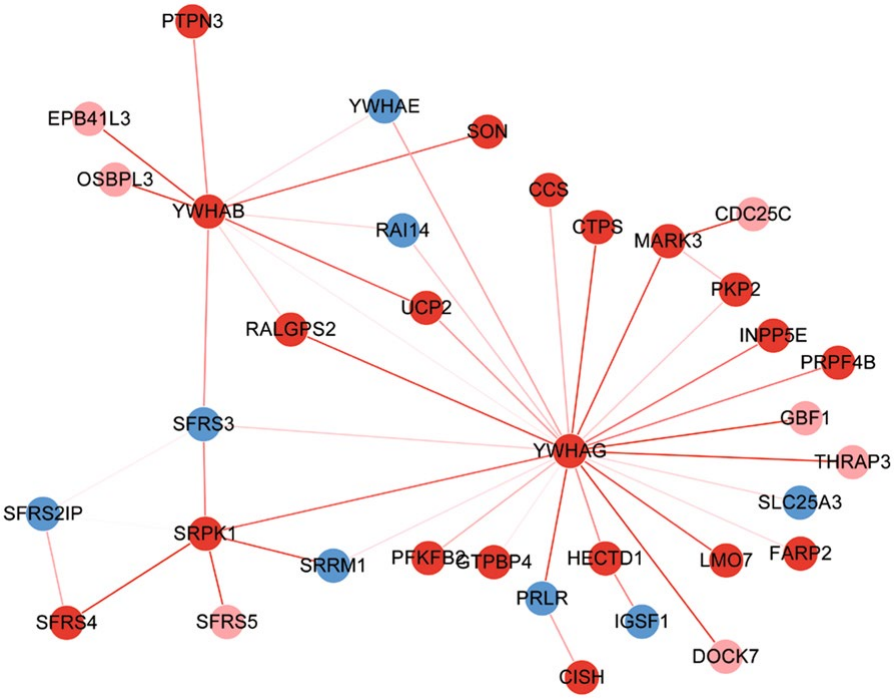
A module with a significantly discriminative score as mDEG + mDEVG + mDECG but not as mDEG. This module has significant discriminative scores of samples from control and case groups, whose DEGs are in *red*, DEVGs are in *blue* and DECGs are in *pink*. Particularly, in this module, SRPK1 and SFRS4 are DEGs; SFRS3 and SFRS21P are DEVGs; meanwhile, and SFRS5 is DECG because it has significantly differential correlation with SRPK1. Obviously, in the conventional differential expression analysis, only SRPK1 and SFRS4 are selected and measured in the downstream analysis, meanwhile other feature genes would be under-estimated.

**Figure 5:**
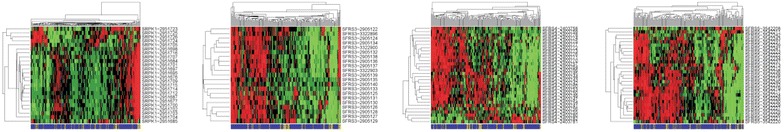
Exon expression profiles of genes related to alternative splicing. For the genes in the differential module related to alternative splicing (AS), their exon expressions have been further investigated by other independent public dataset, because a key factor of the heterogeneity of prostate cancer is just AS mechanism. Each heatmap shows the expression profiles of one gene’s exons on different samples (normal samples are labelled in *yellow*, and disease samples are labelled in *blue*).

A module has been found to have a significantly discriminative score as mDEG + mDEVG + mDECG but not as mDEG. Thus, this module tends to be easily under-estimated in the conventional differential network analysis. This module, as shown in Figure 4, has significant discriminative scores of samples from control and case groups. Particularly, in this module, SRPK1 and SFRS4 are DEGs and SFRS3 and SFRS21P are DEVGs; meanwhile, SFRS5 is DECG because it has significantly differential correlation with SRPK1. Obviously, in the conventional differential expression analysis, only SRPK1 and SFRS4 are selected and measured in the downstream analysis, which will miss much other important differential information. More importantly, these genes/proteins in the differential module all have biological functions related to alternative splicing (AS), and a key factor of the heterogeneity of prostate cancer is just AS mechanism [32]. Thus, we further checked the module genes’ exon expressions by other public dataset [33]. Each heatmap in Figure 5 shows the expression profiles of some genes’ exons on different samples (normal samples are labelled in yellow, and disease samples are labelled in blue). Obviously, these genes have different exon expression patterns in disease samples, and the exons for one gene also have differential expression behaviours.

## Discussion and conclusions

As a benchmark [18], the analysis on a prostate cancer dataset gave strong evidence: (1) the expression variance has additional new differential information comparing to the differential expression; (2) the DEVC-based differential expression network (DEVC-net) has a bi-coloured structure, in which DEVGs are particularly connected as a pathway rather than general hub-centred network; (3) the differential modules from DEVC-net can be quantified by a differential score in single samples, which have improved discriminative ability on phenotypes than the conventional DEGs based methods. Meanwhile, DEVC-net also achieves consistently superior performances on the diabetes dataset (seeing supplementary files).

In fact, the module or gene set based quantification of differential gene expression has been known to have the effect for avoiding the false-positive observation on single genes. Meanwhile, the divergent differential measurements on gene expression (e.g., expression variance and expression covariance) can further extract differential information of gene network/module, and thus the DEVC-net can have strong discriminative ability on phenotypes by combining the power of network inference and its measurements in single samples.

To extract the personalized dysfunctional gene network, DEVC score and its based network analysis DEVC-net were proposed. The gene expression, expression variance and expression covariance all characterize divergent expression patterns involved in the gene network and its modules, which provide interpretable clues on characterizing complex diseases. The differential score DEVC can effectively quantify the differential expressions of a gene network by combining original expression levels (for DEGs), absolute relative expression levels (for DEVGs) and co-expression levels (for DECGs), which extract the discriminative features of the gene network in one sample as the personalized dysfunctional gene network for identifying diseases. As a future topic, it is worth further studying the optimal classification model based on DEVC-net for network biomarker [2] or dynamical network biomarker (DNB) [34, 35], which are necessary to the translational medicine, especially the personalized medicine or precision medicine.

## Additional files

Additional file 1: Additional results. Additional file 2:
**Table S1.** R_pca_MCL.csv: the differential modules mined in prostate cancer dataset. Additional file 3:
**Table S2.** R_T12D_MCL.csv: the differential modules mined in diabetes dataset.
