# Supplementary material for: Unravelling personalized dysfunctional gene network of complex diseases based on differential network model
Source: J Transl Med. 2015 Jun 13;13:189. doi: 10.1186/s12967-015-0546-5 (PMC4467679; doi:10.1186/s12967-015-0546-5)
Supplement: Additional file 1: — Additional results. [file 12967_2015_546_MOESM1_ESM.docx]

**Supplementary**

**Unravelling personalized dysfunctional gene network of complex diseases based on differential network model**

Xiang-tian Yu^1,2^, Tao Zeng^2^, Xiangdong Wang^3,4,*^, Guojun Li^1,*^ and Luonan Chen^2,4,5,*^

^1^ School of Mathematics, Shandong University, Jinan 250100, China

^2^ Key Laboratory of Systems Biology, Shanghai Institutes for Biological Sciences, Chinese Academy of Sciences, Shanghai 200031, China

^3^ Department of Respiratory Medicine, Zhongshan Hospital, Fudan University, Shanghai, China

^4^ Fudan University Center for Clinical Bioinformatics, Shanghai Institute of Clinical Bioinformatics

^5^ School of Life Science and Technology, ShanghaiTech University, Shanghai 201210, China

* Corresponding authors

**S1 A toy model for DEVC**

As a toy model shown in Figure S1, the six genes have two differentially expressed (i.e. g_1_ and g_2_) (Figure S1 (A)), but they cannot group the samples in two pre-defined clusters (labelled as classes *c* and *d*) correctly (Figure S1 (B)). According to the DEVC measurements, the genes g_3_ and g_4_ have differential expression variance and the gene-pairs (e.g. g_5_ and g_6_, noted as g_5_g_6_) have shown differential expression covariance. Replacing the original expression values of these genes to their DEVC-corresponding values, a new expression profile of DEVC-net can be obtained. Such profiles can group samples well (Figure S1 (C)). Thus, DEVC-net is effective to capture the additional differential information except for conventional differential expression, and organize this information in a network manner.

**Figure S1** A toy model for differential expression, expression variance and covariance.

**S2 Another case studies on Diabetes**

The gene expression dataset of diabetes was downloaded from NCBI GEO with access ID GSE9006 [[1](#_ENREF_1)]. It contains 117 blood samples (81 samples of T1D v.s. 36 samples of normal & T2D) with 13785 genes after pre-procession. This data has been analyzed in the study of edge markers [[2](#_ENREF_2)], which validated the usage of differentially correlated gene pairs (i.e. the DECG in DEVC-net). Thus, DEVC-net is expected to integrate the DECG and other differential information to enhance the differential network analysis.

1. **Genes with differential expression variance associated to T1D.** The Top-ranked N genes are chosen (where N is set to 1000) according to the (least) P-value of the significance of differential expression or differential expression variance. Based on these selected DEGs or DEVGs, the samples can be clustered into two groups (81 samples of T1D v.s. 36 samples of normal & T2D [[1](#_ENREF_1)]) by K-means. Again, our numerical experiments (Figure S2) demonstrate that DEVGs can improve the unsupervised grouping of disease samples by themselves or simply combined with DEGs, so that, DEVG and DEG would be complementary kinds of gene features which can capture the differential expression and differential expression variance for single genes respectively. Obviously, the absolute relative expression level is indeed a suitable measurement of DEVGs in single sample; because the absolute relative expression level of DEVGs (DEVG_rel) are better than those of DEGs (DEG_rel), although the original expression level of DEGs (DEG_ori) is better than those of DEVGs (DEVG_ori). Notice that DEVGs show an extremely best performance but also non-robustness, by contrast, the simple combinations (DEG_ori & DEVG_rel) still outperform many other kinds of potential gene signatures slightly but robustly. Besides, 3206 T1D related genes are also extracted from GeneCard; and 243 in Top-1000 DEGs (P=0.1974) are found to be T1D associated, while 261 in Top-1000 DEVGs (P=0.013) are detected. Thus, new gene features (e.g. DEVGs) actually can effectively identify disease genes and catch the potential pathogen mechanism.

**Figure S2** The sample clustering performance on Diabetes dataset based on DEGs and DEVGs and their dissimilar differential measurements.

1. **Bi-coloured differential expression network for T1D.** Combining different gene features, DEVC-net is constructed and its sub-networks like DEG-subnet, DEVG-subnet and DD-subnet are all extracted and investigated their topological structures by network centralities. This analysis (shown in Table S1) supplied repeatable evidence that DEVGs would use long-term interaction to realize their control on biological network (e.g. DEVGs prefer to interact in a form as path rather than hub-centred structure). This is because DEVG-subnet has largest degree centrality but least closeness centrality, compared to the global or other local network structures.

**Table S1** The comparison of network centrality among different sub-networks of DEVC-net on Diabetes dataset

|  | **# Node** | **# Edge** | **Degree** | **Closeness** | **Betweenness** | **Entropy** |
| --- | --- | --- | --- | --- | --- | --- |
| **DEVC-net** | 3148 | 4326 | 0.00087 | 0.11008 | 0.00092 | 7.50557 |
| **DEG-subnet** | 807 | 1191 | 0.00366 | 0.15342 | 0.00373 | 6.21434 |
| **DEVG-subnet** | 149 | 138 | 0.01252 | 0.07346 | 0.01415 | 4.72060 |
| **DD-subnet** | 756 | 862 | 0.00302 | 0.10924 | 0.00415 | 6.18393 |

(A)


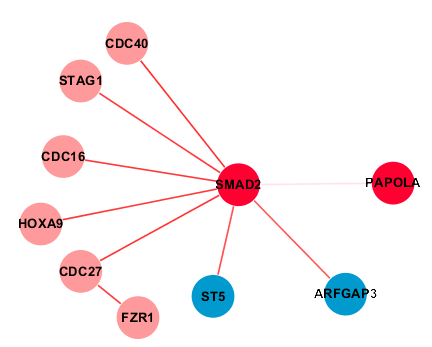


(B)

**Figure S3** Advanced discrimination on phenotypes based on quantified differential expression network / module. (A) The sample-clustering performance based on different module activities (e.g. mDEG, mDEG+mDEVG and mDEG+mDEVG+mDECG respectively). (B) An example module with significantly discriminative score as mDEG+mDEVG+mDECG but not score as mDEG.

1. **The quantified differential expression network and module has advanced discrimination on T1D**. Again, based on the modules identified on DEVC-net by MCL approach, differential score (mDEG+mDEVG+mDECG) and its six kinds of components have been respectively used to classify the binary phenotypes, e.g. T1D and other samples. As seen in Figure S3 (A), (i) when different gene features are separately used to measure modules in differential network, the DEVGs or DECGs would have less discrimination ability on phenotype identification than conventional DEGs but they are still meaningful; (ii) when different gene features are integrated together to quantify network module, especially the differential score combining DEGs, DEVGs and DECGs can effectively promote the clustering performance of downstream analysis (e.g. the clustering accuracy achieves largest and most robust). In addition, a module that is further shown to support DEVC-net actually has more advanced discrimination on phenotypes by integrating the differential network construction and network expression quantification in single samples. From this example in Figure S3 (B), a module consists of ten genes, in which SMAD2 and PAPOLA are DEGs, ST5 and ARFGAP3 are DEVGs, meanwhile, other six genes are DECGs because they almost have significantly differential correlations with SMAD2. Obviously, this module has significantly discriminative score as mDEG+mDEVG+mDECG but not score as mDEG, so that, this module most likely to be under-estimated in conventional differential network analysis. Thus, the proposed DEVC-net has great ability to detect previously non-observed weighted sub-network (e.g. differential module) by making full use of divergent differential expression patterns.

**S3 Additional discussion about the hub-cantered structure**

A low closeness centrality of DEVG-subnet may mean a non hub-centered structure. But its high degree centrality seems to be contradictory. As known, the degree centrality, or most other network centralities usually indicate an average effect. The high degree centrality means many nodes in a network would have high degree. By contrast, hub-centered structure expects only one or very few nodes with extremely high degree than others. In our experimental case, that means it is possible no one or so many nodes with extremely high degree than others, i.e. no node can be thought as a hub with significance. In addition, a simple example can be given as following Figure S4. Such three-node network has average degree 4/3 in condition (A) but 2 in condition (B). Obviously network in condition (A) tends to show a hub-centered structure. Because, in (A), the remove of node 1 will dis-connect remaining nodes 2 and 3. But, in (B), the remove of node 1 will not affect the connection between nodes 2 and 3, which is the same for remove of any other nodes.


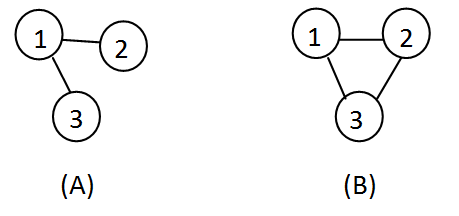


**Figure S4** A sample example about the relation between non-centred structure and high degree centrality

**Reference**

1. Kaizer EC, Glaser CL, Chaussabel D, Banchereau J, Pascual V, White PC: **Gene expression in peripheral blood mononuclear cells from children with diabetes**. *J Clin Endocrinol Metab* 2007, **92**(9):3705-3711.

2. Zhang W, Zeng T, Chen L: **EdgeMarker: Identifying differentially correlated molecule pairs as edge-biomarkers**. *J Theor Biol* 2014.
